# Assessing and predicting protein interactions by combining manifold embedding with multiple information integration

**DOI:** 10.1186/1471-2105-13-S7-S3

**Published:** 2012-05-08

**Authors:** Ying-Ke Lei, Zhu-Hong You, Zhen Ji, Lin Zhu, De-Shuang Huang

**Affiliations:** 1 Tongji University, 1239 Siping Road, Shanghai, P.R. China; 2 Department of Information, Electronic Engineering Institute, Hefei, Anhui 230027, P.R. China; 3 College of Computer Science and Software Engineering, Shenzhen University, Shenzhen, Guangdong 518060, P.R. China; 4 Department of Automation, University of Science and Technology of China, Hefei, Anhui 230027, P.R. China

## Abstract

**Background:**

Protein-protein interactions (PPIs) play crucial roles in virtually every aspect of cellular function within an organism. Over the last decade, the development of novel high-throughput techniques has resulted in enormous amounts of data and provided valuable resources for studying protein interactions. However, these high-throughput protein interaction data are often associated with high false positive and false negative rates. It is therefore highly desirable to develop scalable methods to identify these errors from the computational perspective.

**Results:**

We have developed a robust computational technique for assessing the reliability of interactions and predicting new interactions by combining manifold embedding with multiple information integration. Validation of the proposed method was performed with extensive experiments on densely-connected and sparse PPI networks of yeast respectively. Results demonstrate that the interactions ranked top by our method have high functional homogeneity and localization coherence.

**Conclusions:**

Our proposed method achieves better performances than the existing methods no matter assessing or predicting protein interactions. Furthermore, our method is general enough to work over a variety of PPI networks irrespectively of densely-connected or sparse PPI network. Therefore, the proposed algorithm is a much more promising method to detect both false positive and false negative interactions in PPI networks.

## Background

Protein-protein interactions (PPIs) play a critical role in most cellular processes and form the basis of biological mechanisms. Over the last decade, the development of novel high-throughput techniques, such as yeast-two-hybrid (Y2H), tandem affinity purification (TAP), and mass spectrometry (MS), has resulted in a rapid accumulation of data that provide a global description of the whole network of PPI for many organisms [1]. However, due to the limitations of the associated experimental techniques and the dynamic nature of protein interaction maps, the high-throughput methods are prone to a high rate of false-positives and false-negatives, i.e. protein interactions which are identified by the experiment do not take place in the cell or interacting protein pairs can not be identified by current experiment technology. For example, Y2H screens have false negative rates in the range from 43% to 71% and TAP has false negative rates of 15%-50% [2]. False positive rates for Y2H could be as high as 64% and for TAP experiments they could be as high as 77% [2]. Therefore, the mathematical and computational analysis techniques for assessing the reliability of interactions and predicting new interactions of PPI network are highly desirable.

In the past several years, many computational techniques have been proposed to assess and predict protein interactions. Among them, the network-topology-based methods have attracted extensive attention due to geometric intuition and computational feasibility. The main idea of these approaches is to rank the reliability of an interacting protein pair solely based on the topology of the interactions between the protein pair and their neighbors within a short radius [1]. Examples include interaction generality 1 (IG1) [3], interaction generality 2 (IG2) [4], interaction reliability by alternative path (IRAP) [5,6], Czekanowski-Dice distance (CD-Dist) [7], and functional similarity weight (FSWeight) [8]. More specifically, Saito et al. [3,4] developed two indices called IG1 and IG2, which use the local topology of a pair of proteins to rank their interaction probability. Chen et al. [5,6] introduced a more global measure called IRAP, which is defined as the collective reliability of the strongest alternative path between two proteins. A reliability index called CD-Dist, which is defined as the proportion of interaction partners that two proteins have in common, was originally introduced to predict the protein function by Brun et al. [7]. Similarly, Chua et al. [8] introduced an index called FSWeight that exploits indirect neighbors to predict protein functions.

These network-topology-based methods do yield impressive results on some benchmark data sets. However, there are two main shortcomings of using indices such as IG, IRAP, CD-Dist, and FSWeight for assessing and predicting protein interactions. On one hand, most of these methods are based on a single biological evidence, which makes them hardly gain both a high specificity and a good sensitivity at the same time [3-8]. To reduce the intrinsic false positives and false negatives from a single source, researchers tended to integrate multiple data sources. By using logistic regression (LR) approach, Bader et al. [9] combined multiple heterogeneous biological evidences, including mRNA expression, genetic interactions, and protein domain-domain interaction, to predict the biological relevance of protein-protein interactions obtained from high-throughput screens for yeast. Jansen et al. [10,11] used Bayesian network to integrate four genomic features that are only weakly associated with interaction (i.e., mRNA coexpression, biological function of proteins, coessentiality, and colocalization). Qi et al. [12] developed a random-forest-based technique to combine diverse high-throughput biological data sets, including direct experimental data, indirect high throughput data, and sequence-based data sources, for predicting PPI. Dohkan et al. [13] used an SVM to combine multiple domain effects with other protein features, aiming to improve the accuracy of prediction.

On the other hand, their performance will deteriorate rapidly when the network-topology-based methods are applied to sparse PPI networks [1]. This is because these indices like IG, CD-Dist, and FSWeight are constructed on basis of the connectivity information of original PPI networks (graphs). Obviously, the number of direct and indirect interactions is much lower for sparser networks due to limited connectivity, which inevitably leads to unreliable indices. In order to circumvent the shortcoming that these methods are sensitive to the sparseness of PPI network, some researchers adopted low-dimensional modeling to fit a PPI network [14-19]. For example, Przulj et al. [15,16] represented the given PPI network as a geometric random graph in which nodes correspond to uniformly randomly distributed points in a low-dimensional Euclidean space and edges exist between pairs of nodes in the graph if the corresponding points in the space are close enough (within some radius *δ*) according to the Euclidean distance norm. Fitting the geometric random graphs to the 19 publicly available PPI networks of various organisms indicated that it can successfully discover the geometric structures of these PPI networks. They demonstrated that the technique can be efficiently applied to the denoising of PPI data.

It is well known that proteins generate interactions with each other based on their biochemical and structural properties [20]. Mathematically, we can consider these properties to be dimensions of some abstract metric space. Therefore, each protein can be represented as a point in this metric space, and protein interactions with each other restrict the distribution of these proteins (nodes) to some low dimensional manifold embedded in the high-dimensional unorganized observation space. In this paper, we aim to combine manifold learning theory [21-33] with multiple information integration to develop an effective tool for assessing and predicting protein interactions in a two-stage process. In the first stage, the logistic regression approach is used to combine multiple genomic and proteomic data sources, and infer weighted PPI networks in which each edge (e.g., interaction) is associated with a weight representing the probability of that interaction, whereas, in the second stage, a fast isometric feature mapping (fast-ISOPMAP) algorithm based on manifold learning theory is firstly proposed to seek a low-dimensional manifold embedding of the weighted PPI network (graph), which recasts the problem of assessing and predicting protein interactions into the form of measuring similarity between points in its metric space. The embedding is reasonable if it assigns to nodes of a PPI network a set of point in a low dimensional space such that adjacent nodes in the PPI network correspond to points that are close in the low dimensional space, whereas non-adjacent nodes correspond to points that are further away in the low dimensional space. Given such an embedding, we then assign a topological metric called weighted CD-Dist which indicates the reliability of two proteins to interact with each other, to each protein pair in the PPI networks based on the similarity between the points in the embedding space. The quality of assessing and predicting protein interactions will be evaluated in terms of functional homogeneity and localization coherence of the protein interactions.

## Results

In this section, we firstly quantify the success of embedding PPI network into low dimensional metric space using probability density function and Receiver Operator Characteristic (ROC) curve which are learned from the data given by manifold embedding. The performance of the proposed approach is then evaluated using functional homogeneity and localization coherence of protein interactions from four PPI networks that are derived from various scales and high-throughput techniques, i.e., yeast-two-hybrid (Y2H), tandem affinity purification (TAP), and mass spectrometry (MS).

### The distribution of pairwise distance in embedding space for interactions and non-interactions

In order to quantify the success of embedding PPI network into low dimensional metric space, we learn the following two conditional probability density functions based on original PPI network and its embedding space: *p*(*Distance*|*Interaction*) and *p*(*Distance*|*Non*-*interaction*), where *p*(*Distance*|*Interaction*) is the conditional probability density function which describes the distribution of pairwise distances in the embedding space between pairs of proteins which are known to interact (i.e., form edges in the PPI network) and *p*(*Distance*|*Non*-*interaction*) is the probability density function which describes the distribution of pairwise distances between pairs of proteins which do not interact with each other (non-edges in the PPI network).

**Figure 1:**
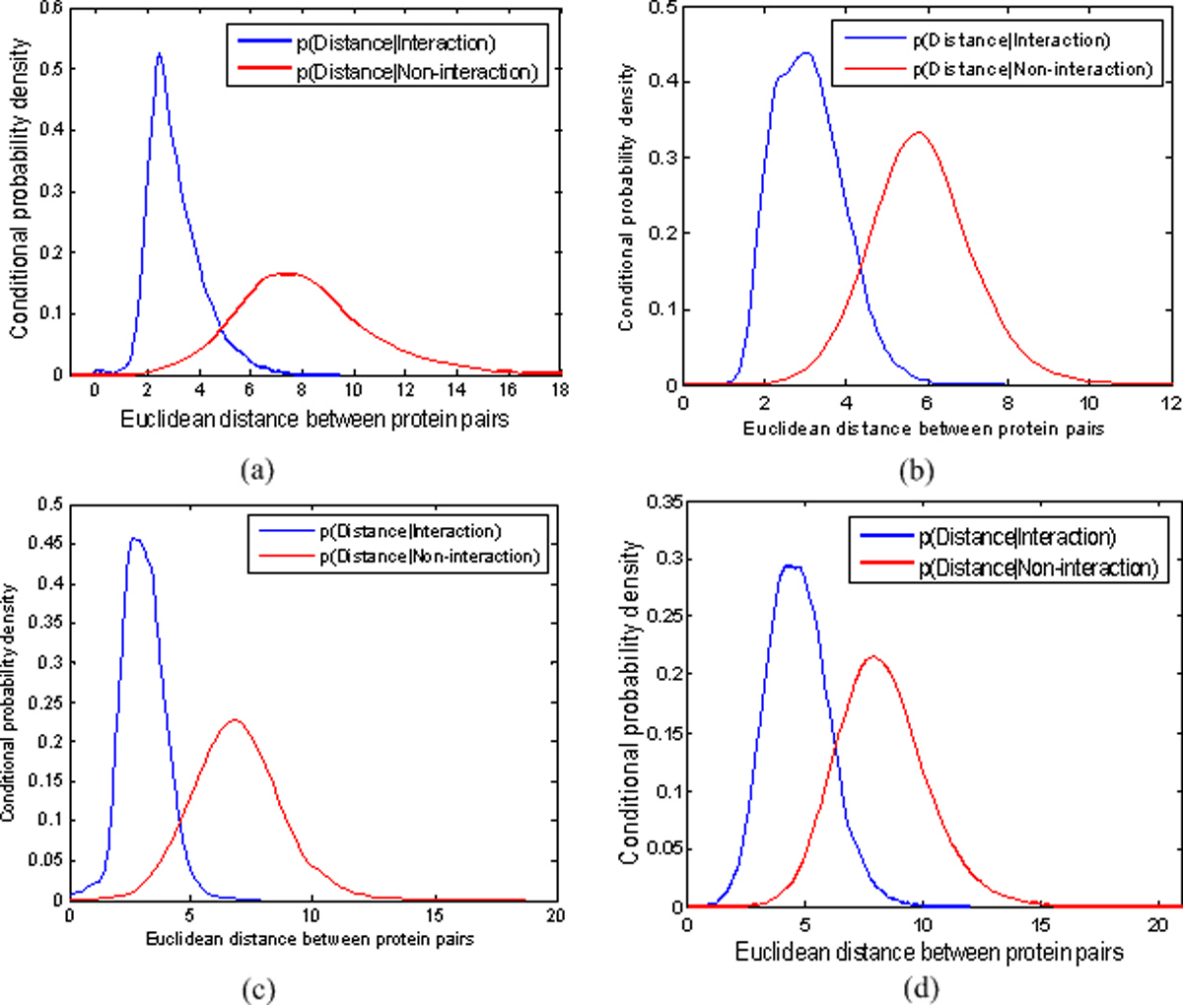
**The conditional probability density functions *p(Distance|Interaction) *and *p(Distance|Non-interaction) *learned from embedding the components of the following four PPI networks into 20-dimentional Euclidean space: (a) Krogan, (b) DIP, (c) Tong, (d) DIP+BioGRID**. The *x *axis denotes the distance between pairs of points in the embedding space and the *y* axis denotes the value of conditional probability density function.

In Figures 1a, b, c, and 1d, we present the conditional probability density functions *p*(*Distance*|*Interaction*) and *p*(*Distance*|*Non*-*interaction*) learned from the data given by embedding the components of the following PPI networks into 20-dimensional Euclidean space: Krogan, DIP, Tong, and DIP+BioGRID. As can be seen, the two functions *p*(*Distance*|*Interaction*) and *p*(*Distance*|*Non*-*interaction*) describe the difference in distance distributions of the interacting and non-interacting protein pairs. More importantly, we find that the interacting protein pairs in the original PPI network are usually very close in its corresponding embedding space while the non-interacting protein pairs in original PPI network are usually far away in the embedding space. It means that PPI network is well represented by embedding it into a low dimensional metric space and the topological structure of the network can be faithfully preserved. In addition, the difference between the two conditional probability density functions proves it is reasonable to classify pairs of nodes into interactions and non-interactions based on the similarity between them in the embedding space.

### Receiver operator characteristic curves for embedding PPI network into metric space

**Figure 2:**
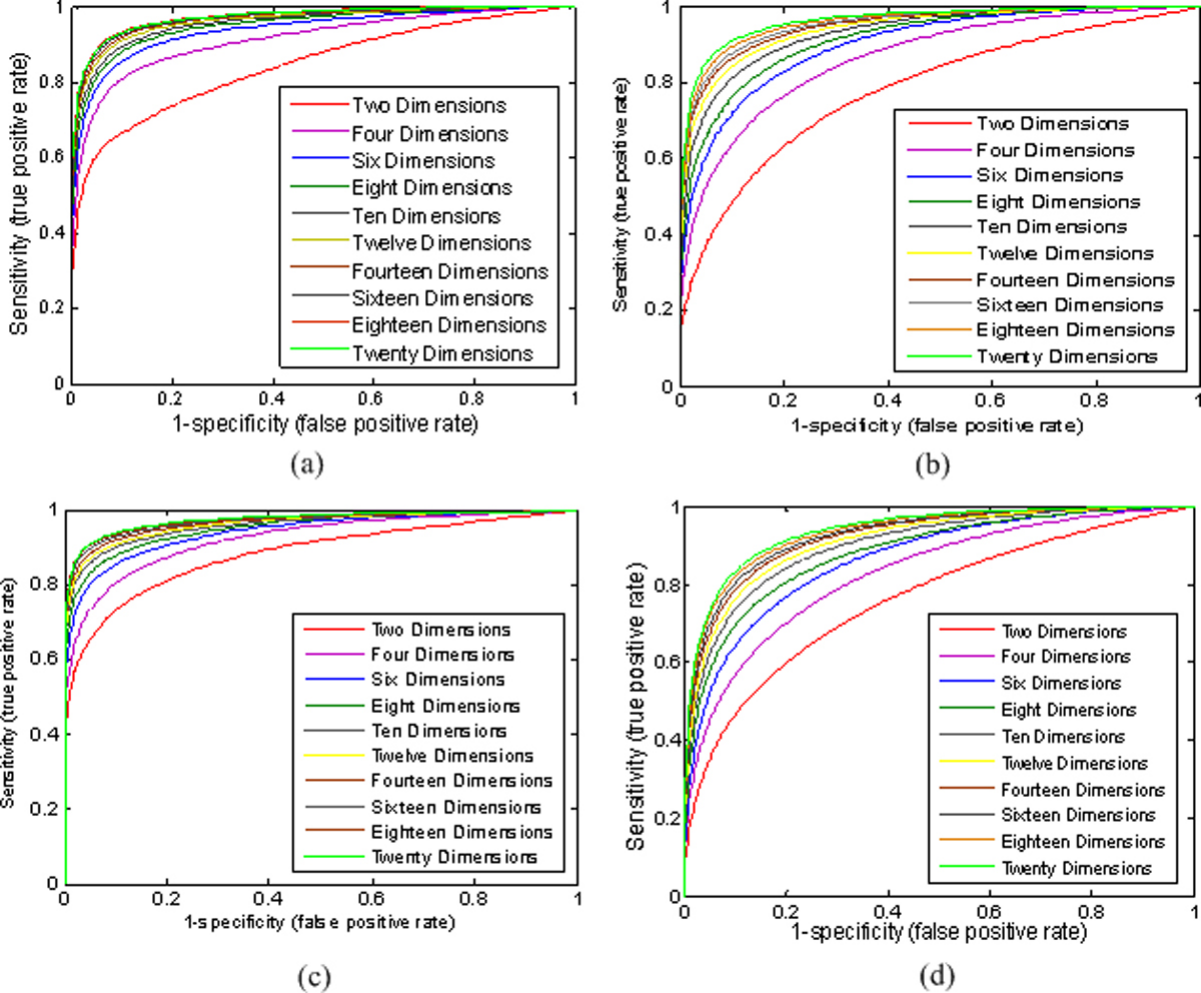
**The ROC curves measuring the ability of recovering the original PPI networks (a) Krogan, (b) DIP, (c) Tong, and (d) DIP+BioGRID using embedding space dimensions of 2 to 20**. The *x *axis of ROC curve is defined as 1-specificity (or false positive rate) and the *y* axis is defined as sensitivity (or true positive rate).

To measure the ability of the proposed manifold embedding method to recover original PPI network, we use a standard ROC curve analysis. Figure 2 demonstrates the ROC curves for the four PPI datasets of *S.Cerevisiae*. For each PPI network, the ten ROC curves for different embedding space dimensions are constructed by varying the distance threshold from 0 to the maximum distance between the points in the corresponding embedding space. Specificity and sensitivity are two commonly used measures of the performance of a binary classification test, and they are defined as follows.

(1) $$specificity=\frac{TN}{TN+FP}\;\text{and}\;sensitivity=\frac{TP}{TP+FN}$$

where TP (True Positive) is the number of true interacting protein pairs which are predicted to be interacting (the distance between point pair in the embedding space is less than a given distance threshold). TN (True Negative) is the number of non-interacting protein pairs that are predicted to be non-interacting (the distance between point pair in embedded space is larger than a given distance threshold). FP (False Positive) is the number of non-interacting protein pairs which are predicted to be interacting, and FN (False Negative) is the number of interacting protein pairs which are predicted to be non-interacting.

It is well known that a ROC curve depicts relative trade-offs between true positive (benefits) and false positive (costs). The best possible ROC curve would contain a point in the upper left corner or coordinate (0, 1) of the ROC space, representing 100% sensitivity (no false negatives) and 100% specificity (no false positives). From Figure 2, we can see that the performances of the ROC curves are encouraging. For example, in Figure 2a, the sensitivity and specificity of ROC curve can reach 92% and 94% respectively when PPI network is embedded into the 20 dimensional space. This corresponds to the false negative rate *β*=1-*sensitivity=*8% and the false positive rate *α*=1-*specificity*=6%. Meanwhile, for dimension 20 of the embedding space, the area under ROC curve is 0.983. It means that the structures of the original PPI networks are faithfully preserved by its corresponding low dimensional spaces. Note that here we regard only those protein interactions in our used PPI datasets as true interactions. However, TAP and Y2H false positive and false negative rates are believed to be at about 64% and 50% correspondingly [2], so using the PPI datasets as the golden standard may underrate the performance of ROC curve. The actual performance of the embedding should be better than what we reported here.

As can be seen from Figure 2, with the decreasing of the embedding space dimension (*d*>2) the performance of the ROC curve is only slightly worse, which means the choice of dimension is not crucial for manifold embedding. Therefore, the PPI network is well modeled by low dimensional embedding metric space and the value of dimensionality does not change the performance much.

### Assessing the reliability of protein interactions

To validate the proposed method for assessing the reliability of protein interactions in the case of embedding into the 20-dimensional space, we systematically compare it with IG [3,4], FSWeight [8], and CD-Dist [7] approaches in term of functional homogeneity and localization coherence on one densely-connected PPI network, DIP+BioGRID [34,35], and three sparse PPI networks, Krogan [36-38], DIP [34], and Tong [39]. For details on these data sets, see Methods, section entitled Data sets under study.

**Figure 3:**
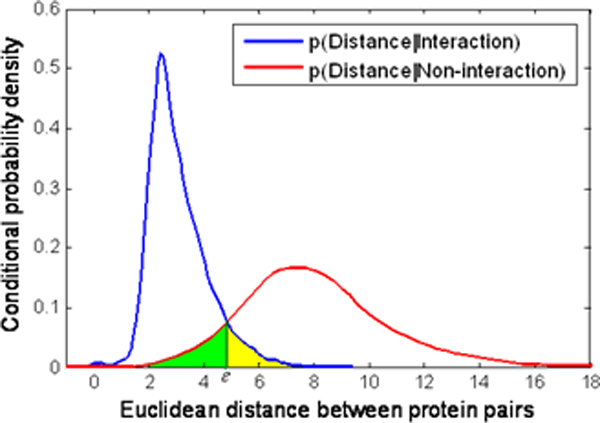
**The choice of the parameter ε**.

For our proposed method, there are two parameters: *d *(the dimension of the embedding space) and *ε *(the similarity cutoff). For the parameter *d*, we found that the proposed method is generally insensitive to the dimension of the embedding space in the last section, therefore, it was empirically set to be 20 in the following experiments. For parameter *ε*, it depends on the distribution of pairwise distance in the embedding space for interactions and non-interactions. Figure 3 illustrates the choice of parameter *ε*. Therefore, for different PPI datasets, a realistic value of *ε *may be different. In addition, it is noticeable that in the original manifold learning algorithms such as isometric feature mapping (ISOMAP), locally linear embedding (LLE), Laplacian eigenmaps (LE), Hessian-based locally linear embedding (HLLE), and local tangent space alignment (LTSA), the neighborhood size *k *is one of the important parameters. The success of these manifold learning algorithms depends greatly on selecting an appropriate neighborhood size. It is well known that how to choose a suitable value of this parameter in constructing a neighborhood graph is an open problem. However, the neighborhood size *k *can be neglected in the context of PPI network dataset because a PPI network can be intuitively represented as a neighborhood graph with proteins as nodes and interactions between the proteins as edges. It obviously contributes to the robustness of our proposed method for assessing and predicting protein interactions.

The strategy of 'guilt by association' provides the evidence that interacting proteins are likely to share a common function and cellular localization [40], which means true interacting protein pairs should share at least a common functional role or they should at least be at a common cellular localization if a pair of proteins to be interacting in *vivo*. Therefore, we utilize the degree of functional homogeneity and localization coherence of protein pairs as the measure to evaluate our method. It is expected that as the true positive interactions increase in the resulting interactome processed by the proposed method, the proportion of interacting proteins with functional homogeneity and localization coherence should increase correspondingly.

In the study, the Gene Ontology (GO) based annotations is used to evaluate the functional homogeneity and localization coherence. The GO is one of the most important ontologies within the bioinformatics community [41]. The three organizing principles of Gene Ontology are cellular component, biological process, and molecular function. Here we used the first taxonomies of the GO terms for localization coherence calculation, and the last two taxonomies of the GO terms for functional homogeneity calculation. The GO terms are organized hierarchically into functional subfamilies. Two different GO terms may have a common parent or a common child in the hierarchy. GO terms at high levels may occur in many genes (or proteins), while GO terms at low levels appear in very few proteins. In our experiment, we just choose those GO terms at middle levels.

### Experiment using the densely-connected PPI network

The experiment is conducted on the DIP+BioGRID dataset. The densely-connected protein interaction network comprises of 72794 non-redundant interactions between 5613 of yeast proteins. Among the 5613 proteins in our densely-connected yeast dataset, 5179 proteins have functional annotations. The number of interactions whose two proteins both have common function annotation is 56639. Therefore, the proportion of interactions with functional homogeneity is 77.8%. About 5165 proteins in this yeast dataset have cellular component annotations. The number of interactions whose two proteins both have common localization annotation is 49469. Therefore, the proportion of interactions with localization coherence in the dataset is 67.9%.

**Figure 4:**
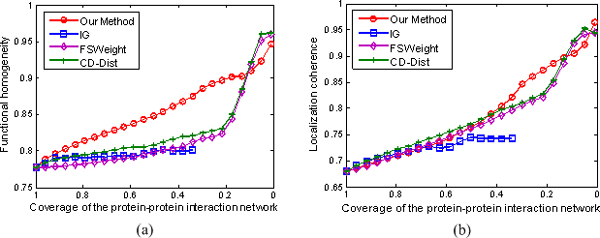
**Comparison of our method, IG, FSWeight, and CD-Dist for assessing the reliability of interactions in term of functional homogeneity and localization coherence**. The comparison is performed by using data on 72794 interactions from the BioGRID database (version 2.0.52) [47] and Database of Interacting Proteins [46]. (a) Functional homogeneity. (b) Localization coherence. The vertical axis is the proportion of interacting protein pairs which share a common function or cellular localization. The horizontal axis is the coverage of the PPI network comparing the original network.

We rank interactions according to their RI values from the lowest to highest, and measure the functional homogeneity and localization coherence by computing the rate of interacting protein pairs with common functional roles and cellular localization. Figure 4 shows the functional homogeneity and localization coherence performance of the interactions in our densely-connected yeast dataset ranked using IG, FSWeight, CD-Dist, and our proposed method. In terms of function homogeneity, it can be seen from Figure 4a that our method is significantly better than IG, FSWeight and CD-Dist methods. For example, more than 90% of the top 20% of the interacting protein pairs ranked according to our method are annotated to have a common cellular role; in contrast, less than 83% of the top 20% of interacting pairs ranked according to FSWeight and CD-Dist are annotated to have a common cellular role. We notice that the proportion of interacting proteins with a common functional role almost doesn't increase in filtered interaction data according to IG method. Similarly, though FSWeight and CD-Dist methods achieve a good performance in terms of localization coherence, our method still exhibits better localization coherence than the other three methods, as shown in Figure 4b. Specifically, as can be seen, our method identifies more interactions that have common cellular localization than any other methods did in the top 20%. The performances of FSWeight and CD-Dist are comparable to it achieved by our method in the top 40%, but their performances are worse than ours after the top 40%. On the whole, our method is clearly better than all other methods for identifying true PPIs. The IG performs the worst among the four methods. Besides, FSWeight and CD-Dist shows a comparable performance.

### Experiment using the sparse PPI networks

As is well known, the real PPI networks are typically very sparse, with average degree of 7 or less [42]. Therefore, in this section, we apply our method to assess the reliability of interactions in three sparse PPI networks, Krogan [36-38], DIP [34], and Tong [39]. Then we systematically evaluate the performance of our method in comparison with IG, FSWeight, and CD-Dist approaches in term of functional homogeneity and localization coherence.

**Figure 5:**
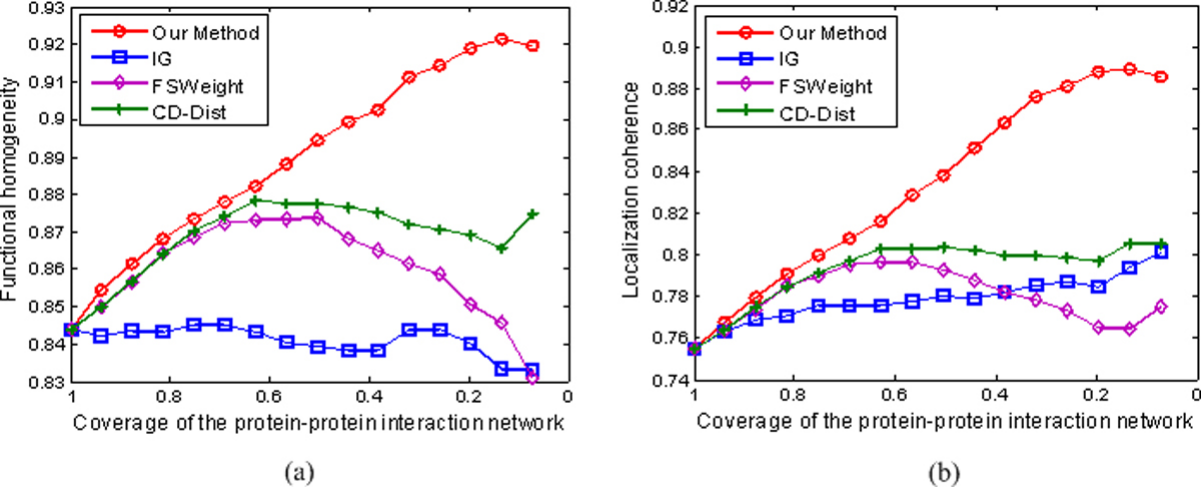
**Comparison of our method, IG, FSWeight, and CD-Dist for assessing the reliability of interactions in term of functional homogeneity and localization coherence**. The comparison is performed by using data on 12934 interactions from the Krogan Lab Interactome Database [43-45]. (a) Functional homogeneity. (b) Localization coherence. The vertical axis is the proportion of interacting protein pairs which share a common function or cellular localization. The horizontal axis is the coverage of the PPI network comparing the original network.

**Figure 6:**
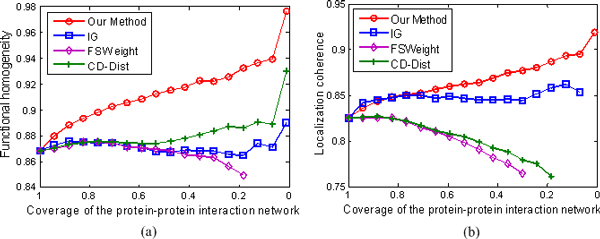
**Comparison of our method, IG, FSWeight, and CD-Dist for assessing the reliability of interactions in term of functional homogeneity and localization coherence**. The comparison is performed by using data on 17173 interactions from the Database of Interacting Proteins [46]. The vertical axis is the proportion of interacting protein pairs which share a common function or cellular localization. The horizontal axis is the coverage of the PPI network comparing the original network.

**Figure 7:**
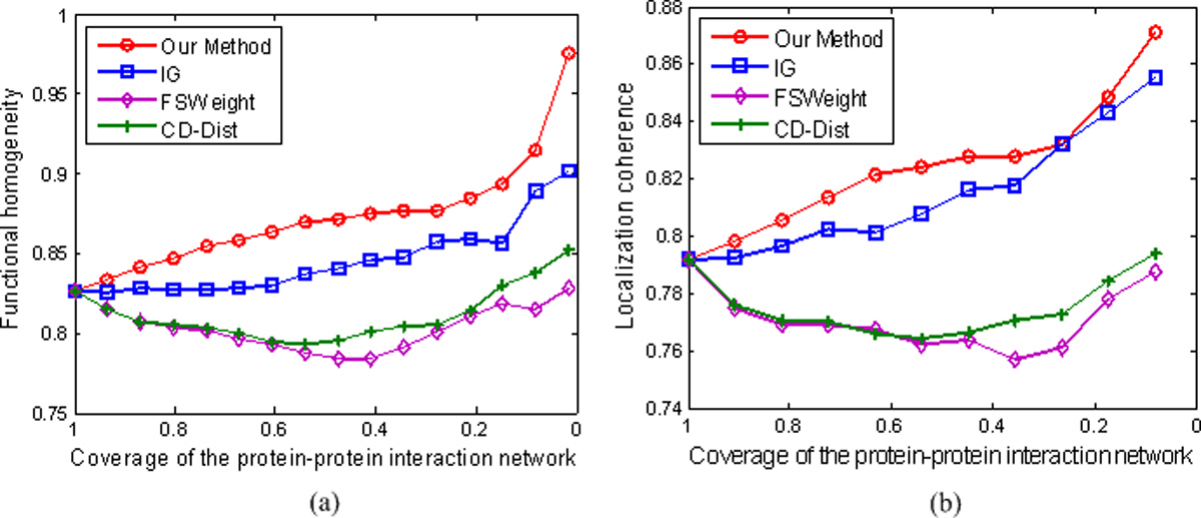
**Comparison of our method, IG, FSWeight, and CD-Dist for assessing the reliability of interactions in term of functional homogeneity and localization coherence**. The comparison is performed by using data on 7622 interactions from Tong et al. [37]. The vertical axis is the proportion of interacting protein pairs which share a common function or cellular localization. The horizontal axis is the coverage of the PPI network comparing the original network.

We rank interactions according to their RI values in the same manner as we did in the last section, and measure the functional homogeneity and localization coherence by computing the rate of interacting protein pairs with common functional roles and cellular localization. The experimental results on the three datasets Krogan, DIP, and Tong are respectively showed in Figures 5, 6, and 7.

In Figure 5a, we show the positive effect of RI value as a filtering measure: as the RI threshold is decreased, the proportion of interacting pairs with common functional roles increases from 84.3% to 92.1%, indicating an increased rate of true positives in the filtered interaction data. For comparison, we also show the performance of IG, FSWeight and CD-Dist in Figure 5a. For the FSWeight and CD-Dist methods, at the beginning, with the decrease in coverage rate, the proportion of interacting pairs with common functional roles increases. However, the trend is not maintained. When they attain their tops 87.4% and 87.8% at 50% and 60% coverages for FSWeight and CD-Dist, respectively, the proportions begin to decrease with the decrease in coverage rate. For IG, the proportion yields with very little increase. Therefore, our method significantly performs the best among the four methods in terms of function homogeneity. Similarly, as shown in Figure 5b, our method exhibits better localization coherence than IG, FSWeight, and CD-Dist. To further demonstrate the effectiveness of our methodology, we also use other two sparse PPI networks of *S.Cerevisiae *derived from various scale and high-throughput technologies. The multifaceted nature of the datasets enables us to perform a more objective comparison of the four algorithms tested. Specifically, we can see from Figures 6 and 7 that the proposed method achieves the best performance in assessing false positive interactions in the sparse yeast dataset--as more interactions which are detected as potential false positive interaction are removed from the interactions, the degree of functional homogeneity and localization coherence in the resulting interactome increases at a faster rate than using other three methods.

Since IG, FSWeight and CD-Dist methods are define purely on basis of the topology of the neighbors of the protein pairs and their formulation implicitly requires the protein pairs being considered to have sufficient number of partners [43], the limited direct and indirect interactions in sparse network lead to their poor performances. However, in our proposed method, some potential connection information, which is very important for methods based on topology to attain good performance, is discovered by preserving local geometry structure. Therefore, we argue that the proposed method is independent on the network sparseness.

### Predicting new protein interactions

In this section, we evaluate our proposed method for predicting new protein interaction, using the same data sets as in assessing the reliability of protein interactions. Because IG method tends to assume interaction between protein pairs to be true, IG may overestimate the reliability for the missing links during the false negative detection process [6]. For example, under the IG method, all missing interactions will be identified as false negatives since they all have the lowest IG values. Moreover, previous works did not report that the IG method is used to detect false negative interactions. Therefore, in the study, we did not use the IG method for false negative prediction. The FSWeight and CD-Dist methods will be compared on these data sets.

**Figure 8:**
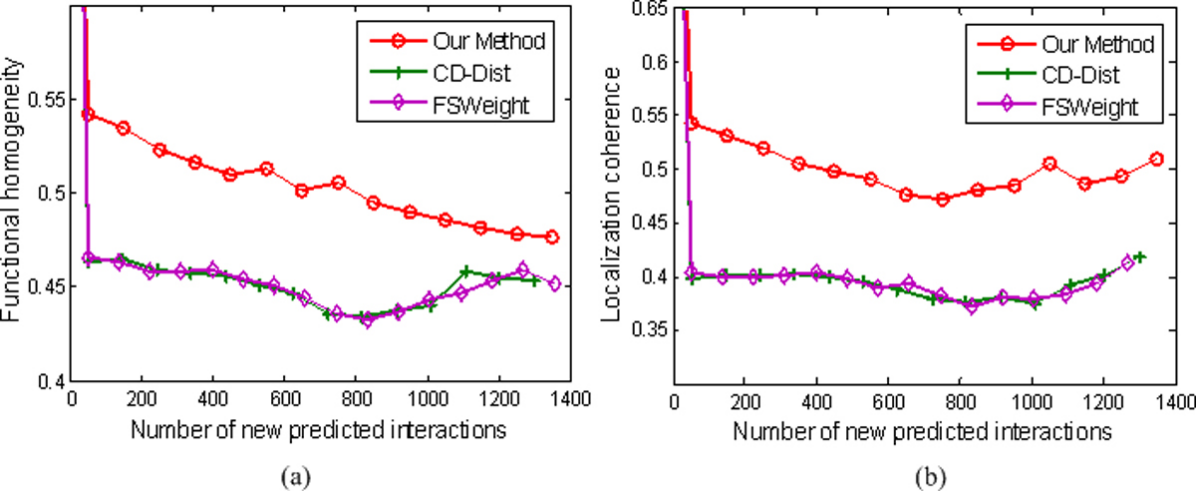
**Comparison of our method, FSWeight, and CD-Dist for predicting new interactions in term of functional homogeneity and localization coherence**. The comparison is performed by using data on 72794 interactions from the BioGRID database (version 2.0.52) [47] and Database of Interacting Proteins [46]. The vertical axis is the proportion of interacting protein pairs which share a common function or cellular localization. The horizontal axis is the number of new predicted interactions.

**Figure 9:**
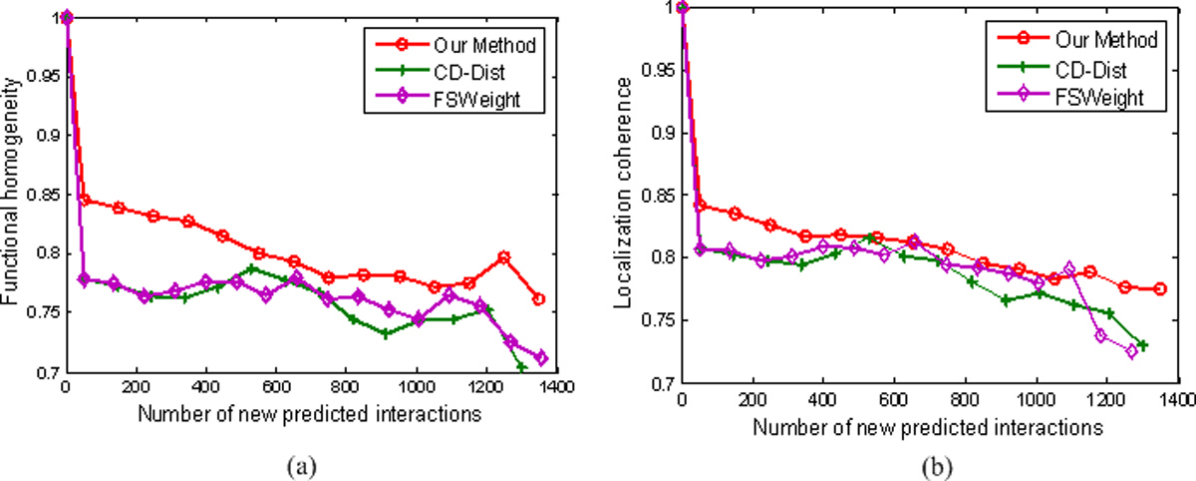
**Comparison of our method, FSWeight, and CD-Dist for predicting new interactions in term of functional homogeneity and localization coherence**. The comparison is performed by using data on 12934 interactions from the Krogan Lab Interactome Database [43-45]. The vertical axis is the proportion of interacting protein pairs which share a common function or cellular localization. The horizontal axis is the number of new predicted interactions.

**Figure 10:**
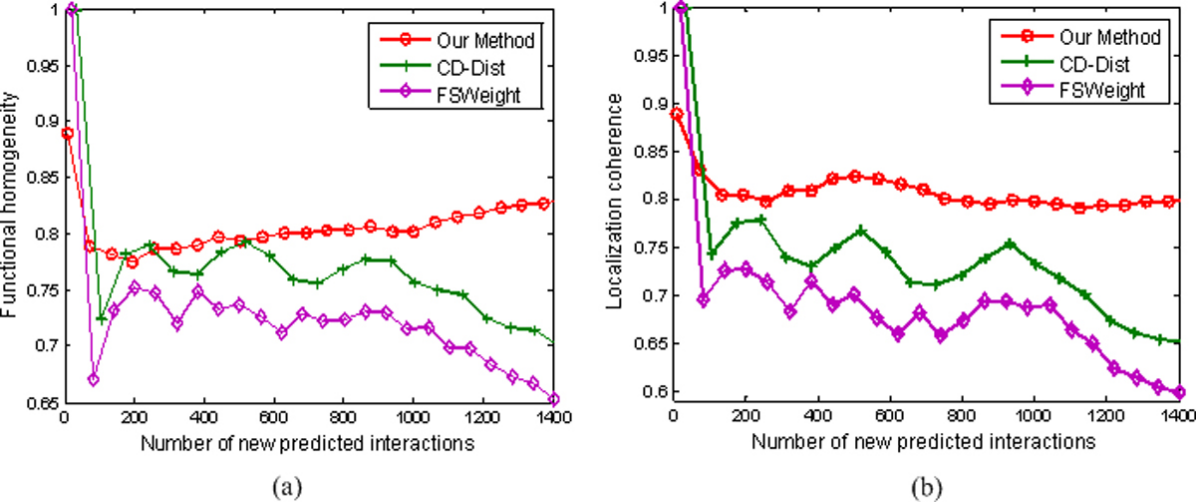
**Comparison of our method, FSWeight, and CD-Dist for predicting new interactions in term of functional homogeneity and localization coherence**. The comparison is performed by using data on 17173 interactions from the Database of Interacting Proteins [46]. The vertical axis is the proportion of interacting protein pairs which share a common function or cellular localization. The horizontal axis is the number of new predicted interactions.

**Figure 11:**
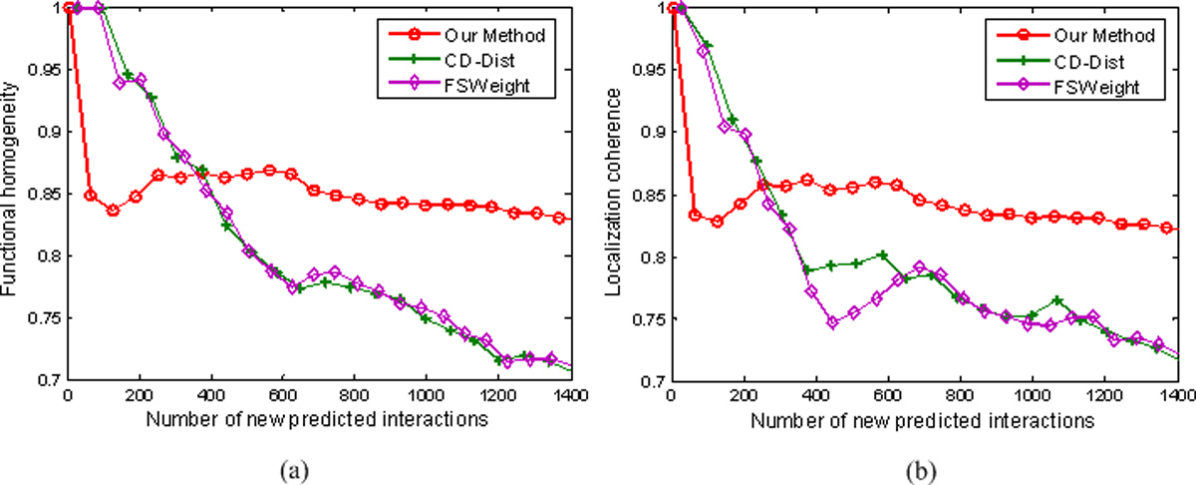
**Comparison of our method, FSWeight, and CD-Dist for predicting new interactions in term of functional homogeneity and localization coherence**. The comparison is performed by using data on 7622 interactions from Tong et al. [37]. The vertical axis is the proportion of interacting protein pairs which share a common function or cellular localization. The horizontal axis is the number of new predicted interactions.

We inspect whether the top new interactions predicted by our method exhibit a higher degree of functional homogeneity and localization coherence than those predicted using other two approaches. The results are illustrated in Figures 8, 9, 10, and 11.

The results in Figure 8 are obtained from the densely-connected PPI network, DIP+BioGRID, which comprises of 72794 non-redundant interactions between 5613 of yeast proteins. The number of protein pairs of proteins that are assigned RI value of 0.059 or higher is about 1400. As can be seen, our method yields satisfactory results. The top new interactions predicted by our method exhibit a higher degree of functional homogeneity and localization coherence than those predicted by other two approaches. FSWeight and CD-Dist perform comparably to each other.

Figure 9 demonstrates the performance of false negative detection on another dataset. The comparison is performed by using data on 12934 interactions from the Krogan Lab Interactome Database. We investigate the biological significance of these PPI prediction results using the Gene Ontology (GO) terms. Clearly, the new interactions predicted by our method are indeed of better quality than the corresponding sets predicted by FSWeight and CD-DIST. For example, as can be seen in Figure 9a, nearly 78% of the top 1400 of the predicted interacting pairs ranked according to our method have a common function, in contrast, less than 75% of the top 1400 of the predicted interacting pairs ranked according to FSWeight and CD-Dist have a common function. Similarly, as shown in Figure 9b, our method exhibits better localization coherence than CD-Dist and FSWeight.

Figure 10 shows the results from the DIP data set of 4875 proteins with 17173 interactions. We set the RI value to be 0.062 and get about 1400 possible pairs of proteins. It can be seen from Figure 10 that FSWeight and CD-Dist attempt to achieve good prediction of new protein interactions, but its results are far from satisfactory. In contrast, our method yields more reliable results than FSWeight and CD-Dist do.

Figure 11 shows an example where the Tong data set of 2171 proteins with 7622 interactions is used. The functional homogeneity and localization coherence of the new interactions predicted by our method in the case of embedding into the 20-dimensional space are illustrated in Figures 11a and 11b. At the beginning, FSWeight and CD-Dist achieve better performance. However, our method outperforms FSWeight and CD-Dist as the number of new predicted interactions is increased. Besides, FSWeight and CD-Dist almost achieve a comparable performance.

## Discussion

It is worthwhile to highlight several aspects of the proposed approach here:

(1) We present a novel network-topology-based approach with information fusion for assessing and predicting protein interactions. It effectively avoids the false positives and false negatives from "single evidence models" such as IG, CD-Dist, and FSWeight.

(2) The purpose of low-dimensional manifold modeling is to represent each node of a PPI network (graph) as a low-dimensional vector which preserves similarities between the node pairs, where similarity is measured by a PPI network (graph) similarity matrix that characterizes certain geometric properties of the data set. By manifold modeling, we make our proposed approach general enough to work over a variety of PPI networks irrespectively of densely-connected or sparse PPI network.

(3) In order to make the ISOMAP algorithm suitable for PPI network datasets, we present a fast ISOMAP algorithm based on minimum set cover (MSC). The success at detecting both new and spurious interactions confirms that the proposed algorithm is able to uncover the intrinsic structural features of PPI network. To our knowledge, this paper is one of the first studies aiming at utilizing manifold learning theory to assess and predict protein interactions.

## Conclusions

In this paper, we have developed a robust technique to assess and predict protein interactions from high-throughput experimental data by combining manifold embedding with multiple biological data integration. The proposed approach first used the logistic regression approach to integrate multiple genomic and proteomic data sources. After obtaining a weighted PPI network, we utilized the fast-ISOMAP algorithm based on manifold learning theory to transform the weighted PPI network into a low dimensional metric space, and then a reliability index which indicates the interacting likelihood of two proteins is assigned to each protein pair in the PPI networks on the basis of the similarity between the points in the embedded space. To the best of our knowledge, this is one of the first studies on assessing and predicting protein interactions which explicitly considers low-dimensional manifold modeling and uses manifold learning theory to embed PPI network into a low-dimensional metric space. The experimental results show our method consistently performs better than the existing network-topology-based methods on both densely-connected and sparse PPI networks, which indicates that the proposed approach is independent on the sparseness of the PPI network and might shed more light on assessing and predicting protein interactions.

Although our experimental results on the four protein interaction data sets demonstrate that our proposed method is insensitive to the dimensionality of the embedding space, the intrinsic dimensionality of data manifold, or degrees of freedom, contributes to capture the inherent attributes hidden in the high-dimensional unorganized observation space. Therefore, how to estimate the intrinsic dimensionality of the PPI dataset is a problem deserving further investigation. In addition, the ISOMAP algorithm requires the analyzed manifold is a convex subset of *R^D^*. Then, the data set must be an open connected subset of *R^D ^*[25]. Therefore, the ISOMAP algorithm can only handle fully-connected PPI networks or the largest connected component of the non-fully-connected ones. But we have to confess that it is very important to assessing and predicting protein interactions for the whole non-fully-connected PPI network. Therefore, we will address this problem and try to build a general framework for assessing and predicting protein interactions based on multimanifold modelling in the near future.

## Methods

### Data sets under study

There are three different types of data used in this paper: 1) gold standard data sets of known interactions (true positives, TPs) and non-interacting protein pairs (true negatives, TNs); 2) genomic and proteomic feature data sets; and 3) protein interaction data sets.

### Gold standard data sets

To provide a measure of assessing the reliability of evidence coming from each information source, the gold standard positive (GSP) and gold standard negative (GSN) data sets, which contain a sufficiently large number of TPs (proteins known to be interacting) and TNs (proteins known to be non-interacting) respectively, are required to constructed. Based on the assumption that proteins belonging to the same complex are likely to interact with each other, information on protein complex membership specified in the MIPS complex catalog is often used to construct the GSP data sets. Unlike positive interactions, it is rare to find a confirmed report on non-interacting pairs. Considering the small fraction of interacting pairs in the total set of potential protein pairs, we use a random set of protein pairs, excluding those interacting pairs that are known, as the GSN data set. In this paper, we collected 12,279 GSP and 19,641,036 GSN protein pairs to train the logistic regression model and compute the model parameters.

### Genomic and proteomic feature data sets

A total of six different types of genomic and proteomic data sets obtained from [12], [44], and [45] were considered. A brief description of these data sets is listed below.

(1) Gene expression. This data set was obtained from publicly available gene expression data [46], and contained 20 gene expression subsets which were recorded under more than 500 conditions (each measuring a time series expression profile). The Pearson correlation for each protein pair, which ranges from -1.0 to 1.0, was calculated.

(2) Essentiality. This data set was derived from ref. [39]. Each protein can be experimentally characterized as either essential (E) or nonessential (N) for survival. Therefore, protein pairs can be categorized three groups: EE indicates that both proteins are essential; NE/EN indicates that one protein is essential while the other is nonessential; and NN indicates that neither of the proteins is essential. The co-essential feature in the data set is a 3-value categorized feature: 2 means EE; 1 means NN, and 0 means NE/EN.

(3) Sequence similarity. This is a quantitative measure of sequence match significance. The data set was generated from the SGD NCBI-BLASTP [47]. We only used the yeast to yeast alignment result from this database. All BLASTP hits obtained with the default parameters that had E-value less than or equal to 0.01 were used and the query protein was excluded from the results.

(4) Transcription factor. This data set, which takes non-negative discrete values, was obtained from a study published by Harbison et al. [48]. Based on the observation that proteins in the same complex are in some cases bound by the same transcription factors [46], transcription factor data can provide interaction information about interaction protein pairs. In our case, we used the S.Cerevisiae transcription-factor (TF) binding data as appeared in [48]. For each pair of proteins, we counted the number of transcription factors that bind to both genes, and used this number as the attribute.

(5) Domain-domain interaction. This data set, which has a value ranging from 0 to 1, was constructed from a study published by Deng et al. [49]. The probabilities of interactions between every pair of domains were inferred by maximum likelihood estimation method [49]. We used the protein interacting probability derived from the above derived domain-domain interaction probability as one attribute.

(6) Homology based PPI. This data set was derived from the SGD NCBI PSI-BLAST hits results [47,50]. We used the 0.001 as a cutoff on the E-value to decide the homology pairs. The final homology feature in the data set is generated by determining if a candidate Yeast protein-protein pair interacts in other species or not. If yes, the feature was the number of times their homology proteins found to interact, otherwise 0.

Table 1 summarizes the six types of genomic and proteomic data sets. In converting evidence sources into feature attributes, summary encoding, in which similar types of experiments are grouped together and provide a single value [10,12,51], was adopted for its simplicity. Among these genomic and proteomic feature data sets, gene expression and Transcription Factor data sets contain 11.1% and 2% missing values, respectively [see Table 1]. We substituted these missing values to the reference state with the mean under that specific experimental condition.

**Table 1:** Characteristics of six genomic and proteomic data

Category	Feature abbreviation	Feature	Number of protein pairs	Number of proteins	Range	Data source
**Genomic data**	GE	Gene expression	19,653,315	6,270	[-1, 1]	[46]
	ESS	Essentiality	667,590	1,156	{2, 1, 0}	[39]
	SEQ	Sequence similarity	7,742	6,270	Real value-Non negative	[47]
	TF	Transcription factor	19,397,106	6,229	Non-negative discrete, most 0	[48]

**Proteomic data**	DD	Domain-domain interaction	125,435	6,359	[0,1]	[49]
	HO	Homology based PPI	177,667	6,270	Non-negative discrete, most 0, 1	[47,50]

### Protein interaction data sets

To demonstrate the effectiveness of our methodology, we have used four PPI datasets of *S.Cerevisiae *derived from various scales and high-throughput technologies. The multifaceted nature of the datasets enables us to perform a more objective comparison of the tested algorithms. Specifically, the first PPI dataset of *S.Cerevisiae *was generated from the Krogan Lab Interactome Database (see http://interactome-cmp.ucsf.edu/). The database contains many protein interactions curated from Krogan et al. [36], Gavin et al. [37], and Collins et al. [38] combined. In this study, the PPI dataset comprises of 12934 non-redundant interactions between 3645 of yeast proteins. The second PPI dataset was downloaded from the Database of Interacting Proteins (DIP) [34], which is a database that documents experimentally determined protein-protein interactions (April, 2007). This data set is composed of 17491 interactions between 4934 yeast proteins. We removed from the dataset any protein with self-interactions and repeated interactions, and the final dataset consists of 17173 interaction pairs involving 4875 proteins. The third dataset was downloaded from ref. [39], and contains 7622 non-redundant interactions between 2171 of the yeast proteins. For the fourth dataset, we first extracted 65536 unique PPI pairs from the BioGRID database (version 2.0.52) [35]. These PPIs were previously identified by using Y2H, TAP, and MS. Then we combined this dataset with our second DIP dataset, and obtained an extremely large PPI dataset which comprises 72794 non-redundant interactions between 5613 of the yeast proteins. A detailed description of these data sets can be found in [34-39]. The characteristics of the data are summarized in Table 2.

**Table 2:** Characteristics of four protein interaction data

Data set	Number of interactions	Number of proteins	Data source
**Krogan**	12934	3645	[36-38]
**DIP**	17173	4875	[34]
**Tong**	7622	2171	[39]
**DIP+BioGRID**	72794	5613	[34,35]

### Algorithm

**Figure 12:**
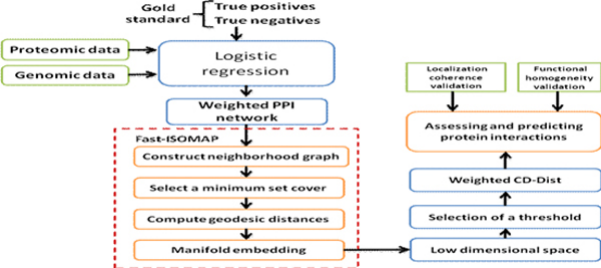
**Framework for the combination of manifold embedding and multiple information integration to assess and predict protein interactions based on the integration of diverse sources**.

The overview framework for utilizing manifold embedding and multiple information integration to assess and predict protein interactions is illustrated in Figure 12. As stated earlier, our approach proceeds in two stages: the first stage to infer a weighted PPI network from the diverse genomic and proteomic data sources and the second to assess and predict protein-protein interactions from the weighted network. In the first stage, we adopt the logistic regression (LR) approach, which uses genomic and proteomic feature data, and the gold standard to train a LR model, i.e., identifying the quantitative effect of each input feature upon protein interactions. Once the LR model has been constructed, it can be used to integrate six data sources to calculate a linkage weight for each protein pair that is connected in the PPI network, and so, a weighted PPI network is obtained. The second stage of our approach maps this network into a low-dimensional feature space by applying a fast manifold embedding algorithm, i.e. fast-ISOMAP algorithm, and then assigns a topological metric called weighted CD-Dist to each protein pair in the PPI network. Thus, although we utilize the LR-based approach to integrate diverse biological information sources, this paper places the emphasis on making use of manifold embedding technique to increase the reliability of protein interactomes based on the integration of genomic and proteomic data sources.

### Logistic regression integration of information sources

Combining evidence from many different sources as features in a supervised learning framework has been proven a successful strategy in reconstructing PPI networks such as yeast [10,12] and human [10,52]. Logistic regression provides a powerful way to integrate information from heterogeneous data sources, due to the fact that the linear relationship between the logit of the explanatory variables and the response one simplifies the integration process and its probabilistic nature enables differences in the contribution of the sources to be taken into account. In addition, the flexibility that it can predict a discrete outcome from a set of variables that may be continuous, discrete, dichotomous, or a mixture of these makes it compatible to any genomic feature data source. For details on the theory of logistic regression, see [9] and [53].

Now, for a protein pair of interest, the linkage weight prediction problem based on their values *u*_1_,*u*_2_,..., *u_n _*in *n *different data sets can be formulated as a two-class classification problem: class +1 (the protein pair is interacting) or class -1 (the protein pair is non-interacting). In this work, logistic regression method is used to infer the probability of an interaction. The basic idea of logistic regression method is introduced briefly as follows.

A general binary regression model takes the form:

(2) $$P\left(y=+1|\beta ,U\right)=\psi \left(\beta^{T}U\right)=\psi \left(\underset{i}{\sum }\beta_{i}u_{i}\right),$$

where the dependent variable *y *denotes the class label, +1 or -1; The explanatory variables *U*=[1,*u*_1_,...,*u_n_*]*^T ^*represents the input feature vector; *β*=[*β*_0_,*β*_1_,..., *β_n_*]*^T ^*is the vector of model parameters; *ψ *is the link function; *P*(*y*=+1|*β*,U) denotes posterior probability of *y*=+1 with certain *β *and *U*. If we use the logistic link function:

(3) $$\psi \left(t\right)=\frac{\text{exp}\left(t\right)}{1+\text{exp}\left(t\right)}.$$

Then, a logistic regression model is expressed in the following:

(4) $$P\left(y=+1|\beta ,U\right)=\frac{\text{exp}\left(\underset{i}{\sum }\beta_{i}u_{i}\right)}{1+\text{exp}\left(\underset{i}{\sum }\beta_{i}u_{i}\right)}.$$

The parameter vector *β *can be calculated by using the maximum likelihood estimation. It can be clearly seen that *β *reflects the size of the contribution of each individual data source. A positive component in *β *means that the corresponding data source increases the probability of the protein interaction, while a negative one means that the corresponding data source decreases the probability of the protein interaction; a large component means that the corresponding data source strongly influences the probability of the protein interaction; while a near-zero component means that the corresponding data source has little influence on the probability of the protein interaction.

### Manifold embedding

Finding a well-fitting null model for weighted PPI networks is a fundamental problem and such a model will provide insights into the interplay between network structure and biological function [18]. In this work, we take an alternative view of manifold embedding to develop an efficient algorithm that models PPI networks. It is based on isometric feature mapping (ISOMAP) [21,54]. Our algorithm embeds the PPI network into a low-dimensional metric space so that nodes of the PPI network can be represented as points in the metric space and the connection information in the PPI network can be indicated by the distance in the metric space, i.e. if two nodes are adjacent in the PPI network, the corresponding points are close enough in the metric space. Thus, the topological structure of the network can be faithfully preserved.

#### Isometric feature mapping

**Figure 13:**
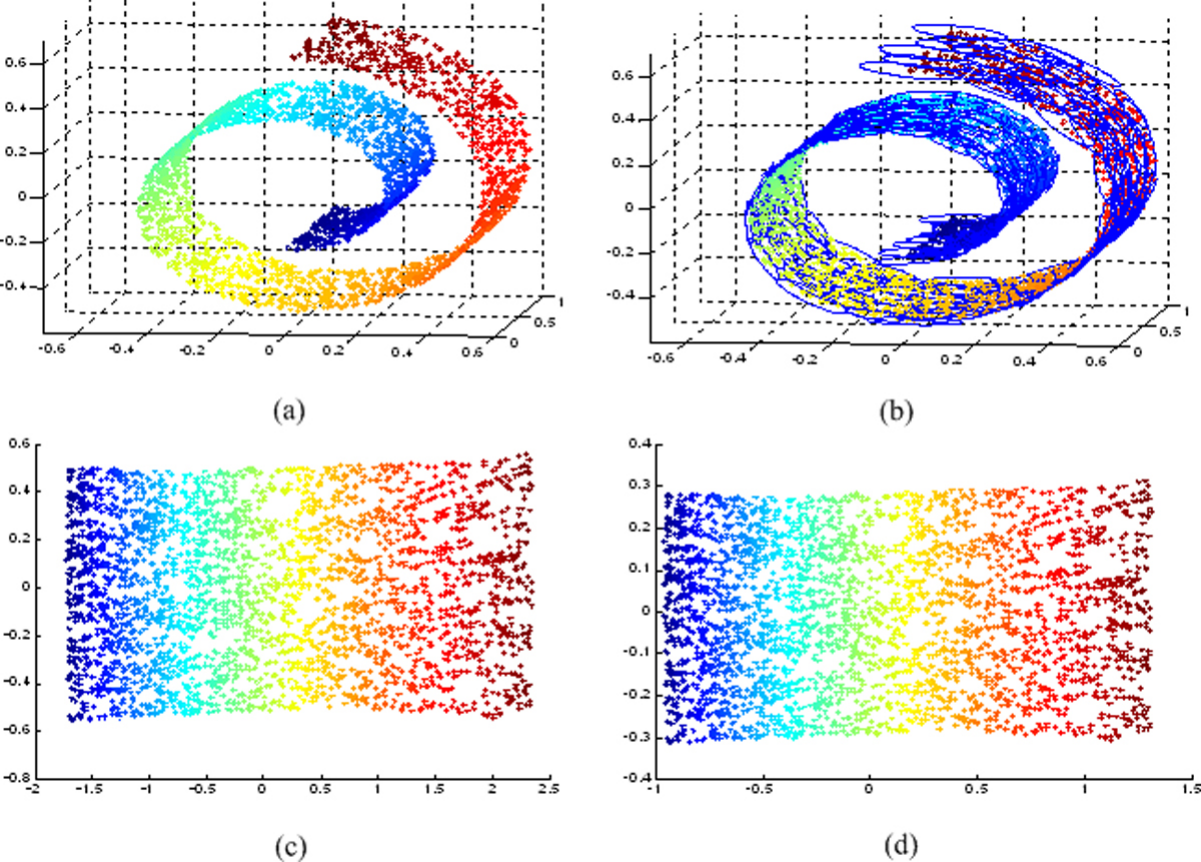
**The 3,000 source data points sampled from a Swiss roll surface and its two dimensional embedding**. (a) Swiss roll data set. (b) Superimposed with minimum subset of neighborhoods. (c) Two-dimensional embedding by ISOMAP. (d) Two-dimensional embedding by fast-ISOMAP.

ISOMAP attempts to find a low-dimensional embedding where the distances between points is approximately equal to the shortest path distances (on a neighborhood graph in the original input space). The power of ISOMAP can be demonstrated by the three-dimensional "Swiss roll" data set in Figure 13a, where points are colored according to their locations on the manifold. When ISOMAP is used to reduce the dimension to two (Figure 13c), the color of the points change gradually, indicating that the representation discovered by ISOMAP faithfully corresponds to the structure of the curved manifold. Although ISOMAP is originally designed for nonlinear dimensionality reduction, here we show that it is suitable for applying to the low-distortion weighted PPI network (graph) embedding problem. The framework of manifold embedding algorithm based on ISOMAP can be summarized as follows.

*Step 1*: Identifying neighborhood graph. A weighted PPI network can be naturally modelled as a weighted undirected neighborhood graph *G*=(*V,E,W*), where the set of vertices *V*={*x*_1_,*x*_2_,...,*x_N_*} are the proteins, and the set of edges *E*={*e_ij_*} indicates neighborhood relationships between the proteins. The weight matrix *W *is obtained by logistic regression integration of multiple heterogeneous biological data sources. The edge *e_ij _*that joins the neighboring proteins *x_i _*and *x_j _*has a weight *w_ij_*, which represents the probability of this interaction. If there is no edge present between a pair of proteins, the corresponding weight is zero. By constructing neighborhood graph, the connectivity and weight information of PPI network can be naturally inherited and the topological structure of PPI network can be faithfully kept.

*Step 2*: Computing geodesic distances. The geodesic distance *d_ij _*between two points *x_i _*and *x_j _*on the manifold can be approximated by the shortest path $d_{ij}^{G}$ between the corresponding vertices in the neighborhood graph. In order to compute the shortest path between every pair of vertices, we use a function of the weight *w_ij _*in lieu of the Euclidean distance. Our experiments suggest that the reciprocal of square root of the weight is a good function for this purpose:

(5) $$d_{ij}^{w}=\left\{\begin{array}{c} 1\;/)\;\sqrt{w_{ij}}\;if\;e_{ij}\in E\\ \infty \;\;\;otherwise \end{array}\right).$$

For neighboring points, $d_{ij}^{w}$ provides a good approximation to the shortest path distance. For faraway points, the shortest path distance can be approximated by adding up a sequence of neighbor-to-neighbor links. These approximations are computed efficiently by finding shortest paths in a graph with edges connecting neighboring data points. The shortest path between every pair of vertices in a graph can be computed efficiently by means of the Dijkstra's or the Floyd's algorithm [55]. If the data points are sampled from a probability distribution that is supported by the entire manifold, then, as the density of data points tend to infinity, it turns out that the estimated $d_{ij}^{G}$ converges to *d_ij _*if the manifold is flat [56].

*Step 3*: Constructing *d*-dimensional embedding. Multidimensional scaling (MDS) [57] is applied to the matrix of the shortest path distance matrix $D^{G}=\left\{d_{ij}^{G}\right\}$ with the purpose of finding an embedding of the weighted PPI network in a *d*-dimensional feature space, so that the geodesic distances between nodes are preserved as much as possible. Let *λ_p _*be the *p*th eigenvalue (in decreasing order) of the matrix *τ*(*D^G^*) (The operator *τ *is defined by *τ*(*D^G^*)=-*HSH*/2, where *S *is the matrix of squared distances $\left\{S_{ij}={\left(d_{ij}^{G}\right)}^{2}\right\}$, and *H *is the "centering matrix" {*H_ij_*=*δ_ij_*-1/*N*}, in which *δ_ij _*is the Kronecker delta function.) and *v_p _*be the *p*th eigenvector. Then the *d*-dimensional embedding can be achieved by $Y=\left[y_{1},...,y_{N}\right]={\left[\sqrt{\lambda_{1}}v_{1},...,\sqrt{\lambda_{d}}v_{d}\right]}^{T}$.

#### Fast isometric feature mapping

Although it has proven to be effective in some benchmark artificial and real world data sets, ISOMAP is limited to data sets with *n≈*2000 sample points and scales poorly to large data sets because it confronts with two computational bottlenecks. One is calculating the *N × N *shortest path distance matrix *D^G^*. Using Floyd's algorithm takes *O*(*N*^3^) time. It can be improved to *O*(*kN*^2^log*N*) by implementing Dijkstra's algorithm. The other is the MDS eigenvalue decomposition, which involves a full *N × N *matrix and has *O*(*N*^3^) time complexity [58]. It is clear that ISOMAP has cubic-time complexity with respect to the number of sample points *N*. When *N *is large, it is infeasible to apply ISOMAP. In this subsection, we present a novel fast ISOMAP based on minimum set cover (MSC) [59] and extend it to the context of assessing and predicting protein interactions.

A prerequisite of the ISOMAP algorithm is to get the *N *neighbourhoods *S*_1_,...,*S_N _*of data points, one for each data point. However, patches derived from all neighborhoods are heavily redundant, and many of them may be ignored with subtle changes to the embedding results. Our objective is to select a minimum subset of the *N *neighborhoods under the constraint that the selected neighborhoods together must cover all data points. Formally, let *X*={*x*_1_, *x*_2_,..., *x_N_*} be a finite set of data points, and let *F*={*S*_1_,..., *S_N_*} be a family of neighborhoods, one for each point in *X*. We say that a neighborhood *S_i_*∈*F *covers data points in *S_i_*. The goal is to compute a minimum-sized subset *C*⊆*F *to cover all data points in *X*, that is, *X*=∪*_S∈C _S*.

This problem is a classical minimum set cover problem which is one of Karp's 21 original NP-complete problems [60]. A typical approximation algorithm to this problem is the greedy algorithm, which iteratively finds a subset that covers the greatest number of remaining uncovered data points. A sketch of such an algorithm is given in Algorithm 1. The set *C *is the cover being constructed. The set *R *is the set of the remaining uncovered data points. The algorithm starts with *R*=*X *and removes from *R *only data points that are covered by a neighborhood *S *that covers as many uncovered data points as possible. The selected neighborhood *S *is added to the cover *C*. When *R *becomes empty, the algorithm terminates, and the set *C *contains a subset of neighborhoods that cover all of *X*.

**Algorithm 1**. Greedy minimum set cover (*X,F*)

1: Initialize *C←*∅,*R*←*X*;

2: **While ***R≠*∅ **do**

3:       Select *S*∈*F *to maximize |*S∩R*|;

4:       *C←C*∪{*S*};

5:       *R*←*R*-*S*;

6: **end while**

**7: **Return (*C*).

Algorithm 1 can be efficiently implemented with time complexity *O*(*∑_S∈F_*|*S*|) [59,60]. We use algorithm 1 to select a minimum subset $C=\left\{S_{a_{1}},...,S_{a_{M}}\right\}$ from the *N *neighborhoods and thereby obtain a landmark point set $L=\left\{x_{a_{1}},...,x_{a_{M}}\right\}$, each landmark point $x_{a_{i}}$ for each neighborhood $S_{a_{i}}$ in *C*. Once a landmak set *L *is chosen, we may compute the shortest paths between each landmark point and all other data points by implementing Dijkstra's algorithm, which leads to a *M × N *distance matrix $D_{M\times N}^{G}$, and not the full *N × N *matrix $D_{N\times N}^{G}$. The landmark multidimensional scaling (LMDS) algorithm [61], which is a computationally efficient approximation to the classical multidimensional scaling (MDS) algorithm, is then applied to the shortest path distance matrix $D_{M\times N}^{G}$ for deriving the low-dimensional embeddings. LMDS mainly includes two steps. Firstly, we apply classical MDS to the landmark points. These second step is a distance-based triangulation procedure, which uses distances to the already-embedded landmark points to determine where the remaining points should go. For a more detailed description of the LMDS algorithm, see ref. [61]. We refer to the proposed algorithm as fast-ISOMAP. Fast-ISOMAP can be much faster than ISOMAP because its main optimization is performed over *M × N *matrix $D_{M\times N}^{G}$, instead of *N × N *matrix $D_{N\times N}^{G}$, where *M × N*. Using the Swiss roll data set in Figure 13a as an example, the data set superimposed with a minimum set cover is shown in Figure 13b. Figure 13d shows the two-dimensional embedding result from fast-ISOMAP. We can see that fast-ISOMAP, as original ISOMAP does, can faithfully preserves the intrinsic geometry structure on this data set. But fast-ISOMAP (which took 2.6897 seconds) is much faster than original ISOMAP (which took 30.7911 seconds).

For PPI networks, where the number of proteins is typically in thousands, the framework of manifold embedding based on fast-ISOMAP can be summarized as

*Step 1*: Identify neighborhood graph *G*=(*V,E,W*).

*Step 2*: Select a minimum subset *C *from *N *neighborhoods *X*_1_,..., *X_N _*using Algorithm 1 and obtain a landmark point set $L=\left\{x_{a_{1}},...,x_{a_{M}}\right\}$.

*Step 3*: Compute the shortest path distance matrix $D_{M\times N}^{G}$ between each landmark point and all other data points given the minimum subset *C*.

*Step 4*: Construct *d*-dimensional embedding by applying the LMDS algorithm to the shortest path distance matrix $D_{M\times N}^{G}$.

### Weighted topological metric

Once the nodes of a PPI network have been embedded to a low-dimensional metric space, we can attempt to characterize the topological property by assigning a suitable reliability index (RI), a likelihood indicating the interaction of two proteins, to each protein pair in PPI network based on the similarities between the points in the embedded space. Here we use the weighted Czekanowski-Dice distance index (weighted CD-Dist) to evaluate the reliability of protein interactions.

Angelelli et al. [62] proposed a new measure called weighted CD-Dist for protein function prediction from protein interaction graphs. The idea of the weighted CD-Dist is based on the observation that protein pairs having many common interaction partners are more likely to have similar physical and biochemical properties and thus they are more likely to be a true positive interacting pairs [8,63-65]. This distance extends Czekanowski-Dice distance [7] to weighted PPI networks. The weighted CD-Dist between protein pair (*u,v*) is defined as:

(6) $$\begin{array}{c} D^{W}\left(u,v\right)\;=w_{uv}\times \frac{\sum_{s\in \Gamma }\left|w_{us}-w_{vs}\right|}{4+\sum_{s\in \Gamma }\left|w_{us}+w_{vs}\right|-2\times w_{uv}}+\\ \;\;\;\;\;\;\;\;\;\;\;\;\;\;\;\left(1-w_{uv}\right)\times \frac{2+\sum_{s\in \Gamma }\left|w_{us}-w_{vs}\right|}{2+\sum_{s\in \Gamma }\left|w_{us}+w_{vs}\right|-2\times w_{uv}}, \end{array}$$

where *w_uv _*is the weight between protein pair (*u,v*) and Γ= Γ(*u*)∪Γ(*v*) in which Γ(*p*) refers to the set that contains *p *and its level-1 neighbors [8]. We can see that the smaller the weighted CD-Dist is, the more likely the two proteins interact with each other.

It is reasonable to use this RI in our study. On one hand, this distance increases the weight of the shared interactors by giving more weight to the similarities than to the differences [7]. On the other hand, weighted CD-Dist makes it possible to take into account the topology information carried not only by the directly interactive proteins, but also by indirectly interactive proteins.

Given a *d*-dimensional embedding of PPI network *Y*=[*y*_1_,...,*y_N_*]∈ℝ*^d × N^*, The procedure of calculating weighted CD-Dist consists of the following steps.

*Step 1*: We create an edge between points *i *and *j *if and only if $d_{ij}^{Y}\leq \varepsilon$, where $d_{ij}^{Y}={∥y_{i}-y_{j}∥}_{2}$ denotes Euclidean distance and *ε*>0 is a similarity cutoff.

*Step 2*: If points *i *and *j *are connected, put $w_{ij}^{Y}=1-d_{ij}^{Y}/d_{\text{max}}^{Y}$ where $d_{\text{max}}^{Y}$ is the maximal Euclidean distance between all pairs of *N *points, otherwise put $w_{ij}^{Y}=0$.

*Step 3*: For each pair of points (*i,j*) in the embedding space, calculate the weighted CD-Dist *D^w^*(*i,j*) based on Eq. (6).

## Competing interests

The authors declare that they have no competing interests.

## Authors' contributions

YL & ZY conceived the algorithm, carried out analyses, prepared the data sets, carried out experiments, and wrote paper. ZJ & LZ was responsible for writing software. All the above actions were supervised by DH.
